# On the Disease of the Hip-Joint, &c.

**Published:** 1837-01

**Authors:** 


### ART. X.

1. *On the Disease of the Hip Joint, with Plates, plain and coloured.* By WM. COULSON, Consulting Surgeon to the London Lying-in Hospital; late Surgeon to the General Dispensary, &c. 4to. pp. 111. *London*, 1837.
2. *On Deformities of the Chest.* By WM. COULSON, Consulting Surgeon to the London Lying-in Hospital, &c. *London*, 1836. Small 8vo. pp. 71. *Four Plates.*

ALTHOUGH these works bear the date of different years, they both appeared in 1836, and are the production of the same author. We shall notice them in the order of their size. Both are very elegant volumes, and do great credit to all concerned in "getting them up,"—the paper-maker, printer, engraver, and even the binder. Mr. Coulson is evidently a man of taste as well as an accomplished surgeon. We hope, however, that his next volume will not follow its predecessor quite so speedily as those before us have respectively done.

In his preface to the work on the hip-joint, Mr. Coulson has made known, as the best possible guarantee for the value of his observations, his connexion with the Royal Sea-bathing Infirmary at Margate, wherein the number of hip-cases are very great. His principal object seems to be, to trace all the subsequent morbid changes which the structures of the hip-joint undergo to a primary affection of the synovial membrane. His dissection of cases in the earliest periods of disease lead him to this conclusion. "These appearances (he says,) lead me to infer that the disease commences most frequently (if not invariably) in the synovial membrane." (*Advertisement*, p. vi.) How far he has succeeded in establishing this principle, the survey of the work which we propose to give will shew.

The First Chapter, "On the Anatomy and Physiology of the Hip-joint," is a suitable and natural introduction to those which follow, but presents nothing of novelty.

In the Second, "On the Causes of the Disease of the Hip-joint," Mr. Coulson thus expresses himself:

> "Many considerations prove that this is a constitutional disease, and that the affection of the hip-joint, and all its metastases, are the mere external demonstrations of a pathological condition, in which extensive function, not a limited locality, are involved." (P. 10.)

He proceeds to consider this as a disease "mainly" of secretion, and produces nine especial reasons on which he grounds his opinion. He condemns the use of local measures, such as "issues, setons, moxæ, &c." as valueless and pernicious:

"For myself I must confess that the signal failure of these means, the exacerbation of nearly every case in which they have been employed, is the circumstance which has led me to these pathological reflections, and to that which seems to me a rational method of treatment." (P. 12.) . . . . . "Previous writers on this subject have ventured to suppose that, when this disease appears in scrofulous persons, it is at least a consequence of constitutional affection. I have endeavoured to show that it is always not a consequence, but a mere external symptom of constitutional disease." (P. 20.)

Our author has truly simplified the subject to some purpose. He has referred the local disease to one local origin, and has transferred the burden of the local disturbance to one constitutional disorder. Thus, when we have just escaped from the thraldom of the one system of treatment for all diseases of the joints, we find ourselves carried back to it by Mr. Coulson. We had ventured to hope that the labours of Sir B. Brodie, and the researches of others who have since investigated these subjects, had emancipated us from the almost empirical systems which formerly prevailed in the treatment of these diseases. Whilst, therefore, we cannot agree with Mr. Coulson in his sweeping condemnation of local treatment, and in his assumption that the constitutional affection is all in all in these diseases, we yet consider his views respecting the constitutional disturbances which accompany them to be very correct. But we cannot persuade ourselves, after a good deal of experience in such cases, that any practical surgeon can place his whole confidence in constitutional measures, or altogether overlook the independent action of local disease. We are quite satisfied that energetic applications to the seat of disease are first to be employed,—no doubt in conjunction with constitutional remedies, which will much aid us in the subsequent progress of the cure.

The whole of the Third Chapter, "On the Pathology of the Hip-joint," is devoted to the consideration of the primary origin of the disease. Mr. Coulson has here collected the opinions of various authors; of these, some differ from him, viz. Albers, Rust, Boyer; some speak of disease as sometimes commencing in the synovial membrane, viz. Wickham, Mayo, Tyrrell, and Key; while Cruveilhier thinks that, in nineteen out of twenty cases, disease commences in the synovial membrane. Mr. Coulson's own opinion does not appear to us to be backed by sufficient authority, or by facts sufficiently numerous and unquestionable, to lead us to consider that he has established his case. On the contrary, we have ourselves witnessed the occasional want of diseased action in the synovial membrane in cases where the bony part of the head of the femur had undergone considerable alteration: and in other joints, especially the knee, our observations have been much more numerous, and lead us to the conclusion that there is no structure which enters into the composition of the joints that may not be the seat of primary disease. We will go further, and say that no surgeon performs his duty who does not ascertain, by the distinguishing symptoms, where the actual seat of disease is, and apply his remedies accordingly. The object of the work of Sir B. Brodie, and subsequently of Mr. Wickham's, has been to set forth these distinctions and their characteristic symptoms.

The observations contained in Chapter IV. "On the Morbid Anatomy of the Hip-joint in this Disease," appear to us to be perfectly correct, but present little or nothing of novelty.

Chapter V. is, "On the Symptoms of the Disease of the Hip-joint." Mr. Coulson has divided the symptoms which occur during the progress of disease of the hip into three stages. The first stage is that in which the altered state of the limb has not arrived, in which the symptoms are only premonitory, such as stiffness, limping, pain; the second stage is that of elongation; the third, that of shortening. It seems to us that the first stage, as marked out by the author, is often undefined; it certainly is often unheeded, and one in which no decided diagnosis can be formed; it blends itself, too, in most cases, so closely with the symptoms of the second stage, that it can hardly be recognized.

The elongation of the limb is, and has been, a stumblingblock to surgery, and no quite satisfactory cause has yet been discovered for this symptom. The explanations commonly given are the four following:—1, that by Mr. Ford, of the filling up of the acetabulum, and consequent pushing out of the head of the femur, whereby the leg is supposed to be lengthened; 2, the descent of the pelvis, and consequently the fall of the limb with it; 3, that suggested by Mr. Wickham, that there exists in reality no actual elongation, but that the limb is rendered rigid by the action of the external rotators, and a deception thereby created in the measurement of the two legs, the inflamed leg being thrown outwards and away from the sound one. The 4th explanation is that recently given by Mr. Tyrrell, which we have noticed at some length in another article. (See Review of *Guy's and St. Thomas's Reports*, p. 146.) We do not by any means consider that Mr. Coulson has set this question at rest: he leans to the first explanation. The shortening of the leg in the third stage is explained by Mr. Coulson as the effect of muscular action. He himself believes, and has brought the evidence of Mr. Liston and Mr. Wickham to prove the rarity of the occurrence of actual dislocation; a circumstance of the highest importance in the treatment of the case. The melancholy ravages produced in the last stages of this complaint are accurately noticed; destruction of the head of the femur and acetabulum, abscess, and the consequent hectic.

Chapter VI. is devoted to the consideration "Of the Diseases with which that of the Hip-joint may be confounded," and is very interesting. It gives information of many of the maladies which may be mistaken for real disease of the hip-joint. These have hitherto not been sufficiently noticed by writers on the subject, and we trust Mr. Coulson, in any future edition, will be enabled to add to the present valuable observations which this chapter contains. As might have been anticipated from the remarks in the beginning of this notice, the author, in Chapter VII. "Of the Treatment of the Disease of the Hip-joint," has mainly directed his attention to the treatment by constitutional means. He does not object to the milder forms of local remedies, but discards all severer measures. On this point we must still remain at issue with Mr. Coulson. From abundant experience, we can state that, in the acutest stage of the disease, the most direct and important benefits are derivable from the use of the seton: it often alleviates the sufferings of the patient in a few hours, and, as far as we are able to perceive, arrests the disease with extraordinary power. Mr. Coulson's directions, in regard to the constitutional treatment, are most judicious. In distinguishing, as examples of inflammation of a tonic character, the cases brought on by cold, external violence, or the metastasis of gonorrhœa, from those of an opposite character spontaneously arising in subjects of a strumous diathesis, Mr. C. has given a practical lesson of great value: his directions, in conformity with this distinction, are clear and important, and merit the particular attention of the surgeon.

Our limits will not allow of our entering into further detail respecting this work; we hope that enough has been said to induce our readers to examine it and judge for themselves. Although we have felt obliged to object to some of the author's views, yet we consider that the treatise does great credit to Mr. Coulson, and recommend it particularly to the young surgeon, as well on account of the valuable matter which it contains as of the importance of the subject of which it treats.

The smaller work of Mr. Coulson, *on Deformities of the Chest*, is much more calculated to advance the knowledge of rational medicine among non-professional readers, than either to instruct the writer's medical brethren or to extend his own professional reputation. It contains nothing that is new or original, and, as a systematic treatise on the subject which it professes to investigate, it must be regarded as both meager and defective. As, however, what it contains is on the whole just, both pathologically and practically, and as we possess in the English language no distinct monograph on the affections treated in it, it may probably be useful to the junior members of the profession. Its proper destination, however,—whether so intended by the author or not we cannot say,—is to the general not the medical reader; and it is one of the too small class of works which can be safely and conscientiously recommended by professional men to the notice of their patients, and the parents and relatives of their patients. Indeed, we do not know a medical volume more calculated to produce beneficial effects on the mind of an intelligent parent; and to all those who are charged with the superintendance of the health of children and young persons, more especially females, we earnestly recommend its perusal. The details it contains of the lamentable evils flowing from the use of stays, in particular, illustrated as these are by striking and intelligible plates of the deformities, can hardly fail to make an impression on any rational mind.

The deformities of the chest treated of by Mr. Coulson are of two kinds, Natural or Spontaneous and Artificial; the former comprehending two varieties, compression or contraction in the lateral diameter, and compression or contraction in the opposite direction, from back to front; the latter embracing all the disfigurements produced by stays or corsets. The principal facts relating to the former class are derived from a memoir of Dupuytren, published in 1825; those relating to the latter are taken chiefly from Sömmering's essay on the Ill Effects of Stays, published as far back as 1788. The first variety of the natural deformity is well known under the name of "chicken or pigeon breast;" in the second, the sternum is depressed and the ribs are laterally prominent. These cases, according to our observation, are almost invariably the consequences of a scrofulous constitution; and we cannot but express our surprise that Mr. Coulson should not at once trace to this general source the principal deformity, as well as the other concomitant morbid conditions, such as chronic enlargement of the tonsils, relaxation and intumescence of the mucous membrane of the air-passages, &c., instead of making the one the consequence of the other. Indeed, a marked defect in the author's pathological views is his failing to recognize, or at least fully to appreciate, the constitutional origin of the diseases which he notices; a defect which necessarily leads to imperfect therapeutic measures. "My trust (he says,) is in other than constitutional remedies." We venture to say that, however valuable and necessary "other remedies" are, no trust can be safely reposed in them without the co-operation of constitutional treatment. The author's mode of viewing this class of cases is the more remarkable, as, in the work already noticed, we had to complain of his views being too exclusively constitutional, both in his pathology and practice.

Mr. Coulson's surgical treatment of these deformities is very judicious; and we quite coincide in his views of the absolute necessity of combining muscular exercise with the manipulations immediately calculated to remove the more prominent deviation from the natural shape. It unfortunately, however, happens that the deformities are often most prevalent at so early an age as to preclude the employment of muscular action.

The following extracts describe the local modes of treatment recommended by Dupuytren, and adopted by Mr. Coulson with slight modification: the interesting and instructive case by Dupuytren with which they conclude (but which we cannot find room for,) places in a striking light the utility of the practice, and the necessity of perseverance in order to ensure its full benefits. In the chicken-breast, the local treatment consists in "frequent pressure upon the sternum from before backwards." The mode of applying this pressure is thus stated by Dupuytren:

"It consists, after the child has been placed sideways, in placing the hand or knee against his back, or, still better, his back against the wall, and placing the palm of the other hand upon the most projecting part of the sternum, and in pressing or pushing the anterior part of the chest towards the posterior part, by alternate movements, which, after some days' practice, accord so well with the movements of respiration, that the little patients, and those who exercise the pressure, soon learn to exercise it during the time of expiration, and to suspend it, so as to allow the breast to develope itself, during the moment of inspiration. During these movements, a sound is heard similar to that made by the air in alternately entering and escaping from a bellows. I have often attentively observed the immediate effects of this exercise: these effects are a flattening of the projection of the sternum, a greater or less bending outwards of the ribs, the momentary return of the chest to a more natural shape, respiration much more strong and perfect than in general, and, when the pressure is removed, the *immediate return* of the parts to their ordinary state, accompanied with a strong inspiration. These pressures should be repeated ten times—a hundred times aday, if it were possible, and continued for several minutes each time: their efficacy will be in proportion to their frequency and duration." (P. 21.)

Mr. Coulson adds,

"The chief difficulty which the practitioner has to struggle with in the treatment of this deformity, is that of impressing on the minds of parents the absolute necessity of perseverance in the use of pressure, and of disposing them to continue with sufficient assiduity a process of which the results cannot for some time be visible. When we must trust to hired nurses, the difficulty, I fear, would be almost insuperable.

Dupuytren accordingly says, 'The practising of these pressures must not be indifferently consigned to any one. A mother's affection alone is capable of the perseverance requisite for success: with this ally, there is scarcely any malformation of the kind we have described that cannot be remedied; and I have seen children who were dreadfully afflicted become eventually strong and well constituted.'

"In the second kind of deformity local pressure may also be made, but in a different direction from that in the former case. In the first kind of deformity, it must be made from before backwards; in this, from side to side. The child or patient must be placed with one side against the wall, and pressure be made either with the hand or the knee against the prominent part of the opposite side, from below upwards. By pressing the ribs upwards, we tilt the sternum forwards, and in some degree imitate the natural action of the parts. The pressure should be made during expiration and suspended during inspiration, as in the former case. Parents are frequently astonished to see the degree of pressure which can be kept up without producing any inconvenience." (P. 23.)

When the child is of sufficient age to use muscular exercise, the following method is recommended by Dupuytren in the pigeon-breast:

"The object is to raise up the sides of the chest, to separate them, to make them turn outward, and finally to restore them to their natural conformation. There is no exercise better adapted for this purpose than that which obliges persons labouring under this malformation to raise a weight suspended to a cord passed through two pulleys, by the aid of their arms and hands, during several hours daily. The end of the cord to be grasped should be fastened to the middle of a lever to be taken hold of by the two hands, the other extremity supporting a weight proportioned to the strength of the individual. The individual, standing upright, or even rising on tiptoe, to reach the lever placed at the extremity of the cord, seizes it with both his hands; and employing the power of the muscles of the forearm, arm, neck, and chest, to bend the head, chest, and body downwards at the same time, must raise the weights at the other extremity of the cord, and alternately employ the flexor muscles to raise the weight, and the extensors to straighten the body." (P. 32.)

Mr. Coulson thinks a simpler and less irksome mode of exercise sufficient:

"I am now satisfied that carrying the arms backward and approximating the scapulæ, without at the same time depressing the ribs by the weight of the body and the action of the abdominal muscles, is all that is requisite. I object, however, to the employment of dumb-bells for that purpose, on account of the jerk which they produce, the involuntary action (that is, beyond a certain extent,) which that implies, and even the danger which it produces; and I deem the Indian exercises, first described in this country by Donald Walker, in his 'Exercises for Ladies,' as greatly preferable to all others, both in these and every other deformity of the chest. They raise up the ribs and sternum, without the slightest counteracting tendency to depress them, and they give the fullest expansion to the chest. By these various means, if properly persevered in, the chest returns to its natural shape, and the whole system becomes invigorated. In the treatment of the Depressed Sternum, I strongly advise my patients to carry the arms back, at right angles to the body, or as far as they can; and to desist, as much as possible, from stooping. And here also I think the Indian exercises the most efficacious; for it is evident that whatever both raises and expands, or naturally rounds, the chest, at once elevates the depressed sternum, and depresses the elevated one." (P. 34.)

The second part of Mr. Coulson's work is, as we have already stated, taken almost entirely from Sömmering's essay, and contains a most terrific exposition, somewhat exaggerated doubtless, of the ill effects of stays and tight-lacing on the female economy. Copies are also given of the pictorial illustrations of the original author, exhibiting the inroads made on the natural shape by this Procustean invention. Some years since we had these engraved on wood, to accompany some extracts from a valuable article on Physical Education, by Dr. Barlow, of Bath, which were published in the Penny Magazine; and, from the extensive use that has been since made of them by writers on Hygiène in this and other countries, we are led to believe that their appearance in that journal has been productive of considerable benefit. We doubt not but the finer copies of them in Mr. Coulson's book will greatly tend to the same desirable end: they ought to be hung up in every nursery and girls' school in the kingdom.

---

